# Supplementary material for: Coordinative metabolism of glutamine carbon and nitrogen in proliferating cancer cells under hypoxia
Source: Nat Commun. 2019 Jan 14;10:201. doi: 10.1038/s41467-018-08033-9 (PMC6331631; doi:10.1038/s41467-018-08033-9)
Supplement: Supplementary file 1 — Reporting Summary [file 41467_2018_8033_MOESM1_ESM.pdf]

nature research

Corresponding author(s): Binghui Li

Reporting Summary

Nature Research wishes to improve the reproducibility of the work that we publish. This form provides structure for consistency and transparency in reporting. For further information on Nature Research policies, see Authors & Referees and the Editorial Policy Checklist.

Statistical parameters

When statistical analyses are reported, confirm that the following items are present in the relevant location (e.g. figure legend, table legend, main text, or Methods section).

n/a	Confirmed	
☐	☒	The exact sample size (n) for each experimental group/condition, given as a discrete number and unit of measurement
☒	☐	An indication of whether measurements were taken from distinct samples or whether the same sample was measured repeatedly
☐	☒	The statistical test(s) used AND whether they are one- or two-sided<br>*Only common tests should be described solely by name; describe more complex techniques in the Methods section.*
☒	☐	A description of all covariates tested
☒	☐	A description of any assumptions or corrections, such as tests of normality and adjustment for multiple comparisons
☐	☒	A full description of the statistics including central tendency (e.g. means) or other basic estimates (e.g. regression coefficient) AND variation (e.g. standard deviation) or associated estimates of uncertainty (e.g. confidence intervals)
☒	☐	For null hypothesis testing, the test statistic (e.g. F, t, r) with confidence intervals, effect sizes, degrees of freedom and P value noted<br>*Give P values as exact values whenever suitable.*
☒	☐	For Bayesian analysis, information on the choice of priors and Markov chain Monte Carlo settings
☒	☐	For hierarchical and complex designs, identification of the appropriate level for tests and full reporting of outcomes
☒	☐	Estimates of effect sizes (e.g. Cohen's d, Pearson's r), indicating how they were calculated
☐	☒	Clearly defined error bars<br>*State explicitly what error bars represent (e.g. SD, SE, CI)*

Our web collection on statistics for biologists may be useful.

Software and code

Policy information about availability of computer code

Data collection	No specific software was used for data collection.
Data analysis	Microsoft Office Excel 2007 and GraphPad PRISM 7.0 were used for data analysis.

For manuscripts utilizing custom algorithms or software that are central to the research but not yet described in published literature, software must be made available to editors/reviewers upon request. We strongly encourage code deposition in a community repository (e.g. GitHub). See the Nature Research guidelines for submitting code & software for further information.

Data

Policy information about availability of data

All manuscripts must include a data availability statement. This statement should provide the following information, where applicable:

- Accession codes, unique identifiers, or web links for publicly available datasets
- A list of figures that have associated raw data
- A description of any restrictions on data availability

The data that support the findings of this study are available from the corresponding author upon reasonable request.

Field-specific reporting

Please select the best fit for your research. If you are not sure, read the appropriate sections before making your selection.

[x] Life sciences [ ] Behavioural & social sciences [ ] Ecological, evolutionary & environmental sciences

For a reference copy of the document with all sections, see nature.com/authors/policies/ReportingSummary-flat.pdf

Life sciences study design

All studies must disclose on these points even when the disclosure is negative.

Sample size	All the experiments were performed using sample sizes based on standard protocols in the field. No statistical methods were used to predetermine sample sizes.
Data exclusions	No data was excluded from the study.
Replication	All biological experiments were carried out under clearly defined and standard conditions and were repeated at least twice whenever possible. All replication attempts were successful.
Randomization	Sample groups were allocated randomly.
Blinding	In vivo experiment was done by a scientist blinded to the treatment schedule. The in vitro experiments were not carried out blinded because metabolite profiling experiments bias is not possible.

Reporting for specific materials, systems and methods

Materials & experimental systems

n/a	Involved in the study	
[x]	[ ]	Unique biological materials
[ ]	[x]	Antibodies
[ ]	[x]	Eukaryotic cell lines
[x]	[ ]	Palaeontology
[ ]	[x]	Animals and other organisms
[x]	[ ]	Human research participants

Methods

n/a	Involved in the study	
[x]	[ ]	ChIP-seq
[x]	[ ]	Flow cytometry
[x]	[ ]	MRI-based neuroimaging

Antibodies

Antibodies used

Second antibodies:Goat anti-rabbit IgG (P/N 926-68071) and goat anti-mouse IgG (P/N 926-32210) were obtained from Li-Cor (USA). The final concentration of the secondary antibodies used was 0.1 μg/ml (1:10000 dilution).
Primary antibodies: β-Actin (Cat#60008-1, 1:5000 dilution), GAPDH (Cat# 60004-1-lg, 1:5000 dilution),GOT1 (Cat#14886-1-AP, 1:1000 dilution), DHODH (Cat#14877-1-AP, 1:1000 dilution), HIF1α (Cat#20960-1-AP, 1:1000) and UMPS (Cat#14830-1-AP, 1:1000) were purchased from Proteintech (USA). CAD (Cat#sc-376072, 1:1000) from Santa Cruz(USA), pCAD (Ser1859) (Cat#70307, 1:1000) from Cell Signaling Technology and GS(Cat#ab176562, 1:1000) from abcam.

Validation

Goat anti-rabbit IgG (Li-Cor,P/N 926-68071) ;validation https://www.licor.com/bio/products/reagents/secondary_antibodies/irdye_680rd.html
goat anti-mouse IgG (Li-Cor,P/N 926-32210) ;validation https://www.licor.com/bio/products/reagents/secondary_antibodies/irdye_800cw.html
β-Actin (Proteintech ,Cat#60008-1, 1:5000 dilution); validation http://www.ptgcn.com/products/ACTB-Antibody-60008-1-Ig.htm
GAPDH (Proteintech ,Cat# 60004-1-lg, 1:5000 dilution); validation http://www.ptgcn.com/products/GAPDH-Antibody-60004-1-Ig.htm
GOT1 (Proteintech ,Cat#14886-1-AP, 1:1000 dilution); validation http://www.ptgcn.com/products/GOT1-Antibody-14886-1-AP.htm
DHODH (Proteintech ,Cat#14877-1-AP, 1:1000 dilution) ; validation http://www.ptgcn.com/products/DHODH-Antibody-14877-1-AP.htm
HIF1α (Proteintech,Cat#20960-1-AP, 1:1000); validation http://www.ptgcn.com/products/HIF1A-Antibody-20960-1-AP.htm
UMPS (Proteintech,Cat#14830-1-AP, 1:1000); validation http://www.ptgcn.com/products/UMPS-Antibody-14830-1-AP.htm
CAD (Santa Cruz,Cat#sc-376072, 1:1000) ; validation https://www.scbt.com/scbt/zh/product/cps2-antibody-f-6?requestFrom=search

pCAD (Ser1859) (Cell signaling,Cat#70307, 1:1000) ; validation https://www.cst-c.com.cn/products/primary-antibodies/phospho-cad-ser1859-d5o6c-rabbit-mab/70307?site-search-type=Products&N=4294956287&Ntt=pcad+%28ser1859%29&fromPage=plp
GS(abcam,Cat#ab176562, 1:1000) ; validation https://www.abcam.cn/glutamine-synthetase-antibody-epr13022b-ab176562.html

Most of the listed antibodies have been used in our laboratory for many years and have been carefully validated using shRNA kd approach to achieve knockdown cells.
Many antibodies were also validated by the provider as stated on their website.

Eukaryotic cell lines

Policy information about cell lines

Cell line source(s)	MCF-7, A549, HeLa, HCC-LM3, SGC-7901 and 4T1 cells were obtained from ATCC.
Authentication	Authentication was not performed as none of the cells used have been listed in the commonly misidentified lines.
Mycoplasma contamination	All cell lines tested negative for mycoplasma contamination.
Commonly misidentified lines (See ICLAC register)	No misidentified lines were used in this study.

Animals and other organisms

Policy information about studies involving animals; ARRIVE guidelines recommended for reporting animal research

Laboratory animals	Female nude mice (4–5 weeks, 19–20 g) were purchased from the Experimental Animal Center of Nanjing Biomedical Research Institute at Nanjing University.
Wild animals	The study did not involve wild animals.
Field-collected samples	The study did not involve samples collected from the field.
