# Supplementary material for: Coordinative metabolism of glutamine carbon and nitrogen in proliferating cancer cells under hypoxia
Source: Nat Commun. 2019 Jan 14;10:201. doi: 10.1038/s41467-018-08033-9 (PMC6331631; doi:10.1038/s41467-018-08033-9)
Supplement: Supplementary file 2 — Supplementary Information [file 41467_2018_8033_MOESM2_ESM.pdf]

Coordinative metabolism of glutamine carbon and nitrogen in proliferating cancer cells under hypoxia

Yuanyuan Wang[1,]*, Changsen Bai[1,]*, Yuxia Ruan[1,]*, Miao Liu[1], Qiaoyun Chu[2], Li Qiu[1], Chuanzhen Yang[2] and Binghui Li[1,2]

[1]Department of Cancer Cell Biology, Tianjin's Key Laboratory of Cancer Prevention and Therapy, National Clinical Research Center for Cancer, Tianjin Medical University Cancer Institute and Hospital, Tianjin 300060, P. R. China.

[2]Department of Biochemistry and Molecular Biology, Capital Medical University, Beijing 100069, P.R. China.

*Co-first author

Correspondence: bli@ccmu.edu.cn

Supplementary Figures

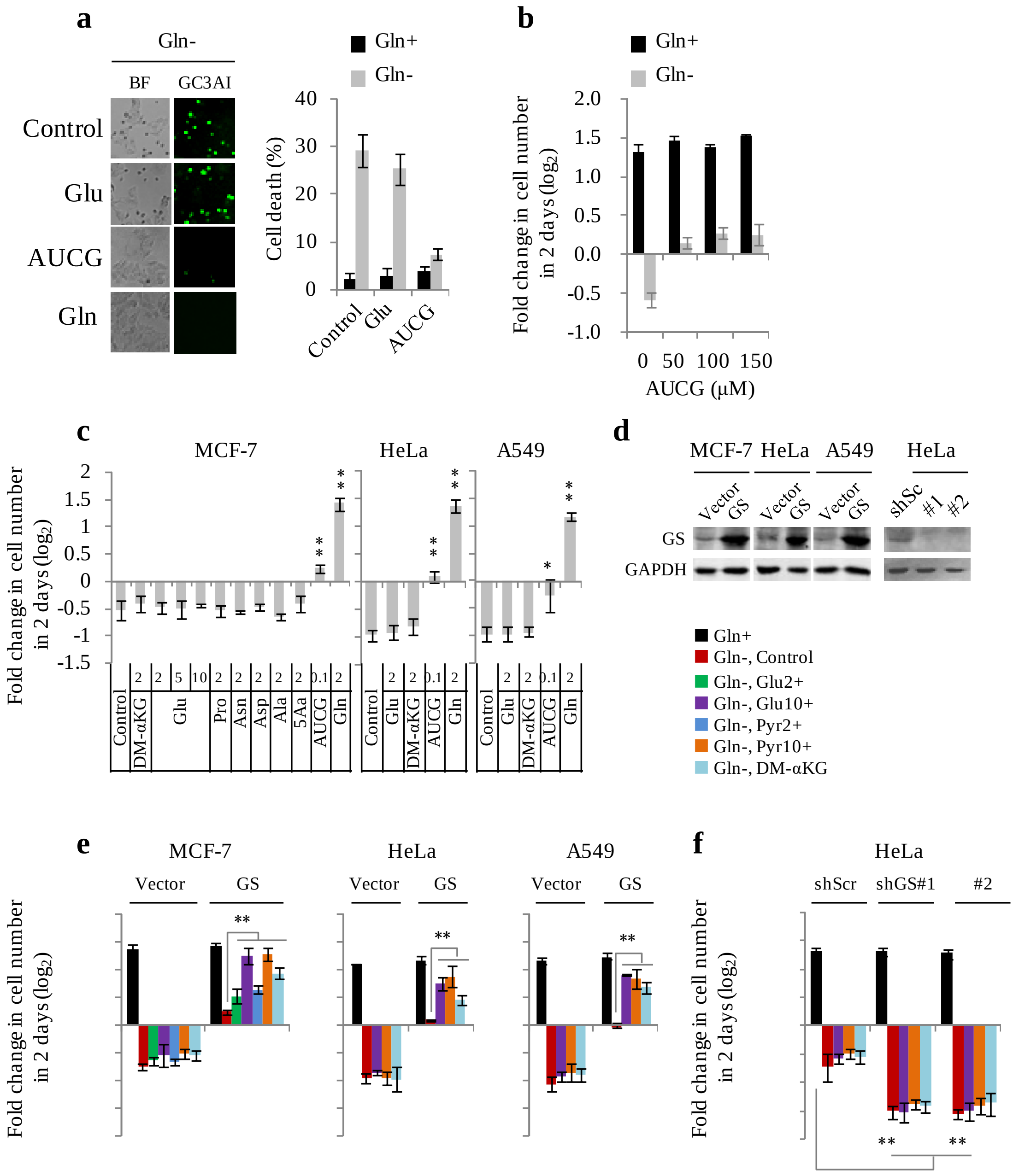

Supplementary Figure 1. Glutamine nitrogen is required for cell proliferation. (a) Cell death in MCF-7 cells cultured with the glutamine-contained or –free medium supplied with glutamate (2 mM), AUCG (0.1 mM) or glutamine (2 mM, as the control) for 48h. Left panel, fluorescent images of MCF-7 cells expressing GC3AI, a caspase-3/7 activation indicator. Green fluorescence indicates apoptosis. Right panel, quantification of cell death of MCF-7/GC3AI cells based on the fluorescent cells. (b) Proliferation of MCF-7 cells in the absence or presence of glutamine (2 mM), with medium being supplemented with different concentrations of nucleosides, including adenosine (A), uridine (U), cytidine (C) and guanosine (G). (c) (a) Proliferation of MCF-7, HeLa and A549 cells cultured with the glutamine-free DMEM medium for 48h in the presence of dimethyl-α-ketoglutarate (2 mM), glutamate (2, 5 or 10 mM), proline (2 mM), asparagine (2 mM), asparatate (2 mM), alanine (2

mM), five non-essential amino acids (2 mM of glutamate, proline, asparagine, asparatate and alanine), nucleosides (0.1 mM of uridine, cytidine, adenosine and guanosine) and glutamine (2 mM, as the control). (d) Western blot of lysates from MCF-7, HeLa and A549 cells expressing empty vector, GS or shRNA against GS. (e) Proliferation of MCF-7, HeLa and A549 cells expressing empty vector or GS cultured with the glutamine-free medium supplemented with nutrients as indicated. (f) Proliferation of MCF-7/shScramble and MCF-7/shGS cells cultured with the glutamine-free medium supplemented with nutrients as indicated. All cultures were supplied with 10% dialyzed serum. Values are the means ± SEM of triplicate experiments. *$p < 0.05$, **$p < 0.01$ (Student's *t*-test).

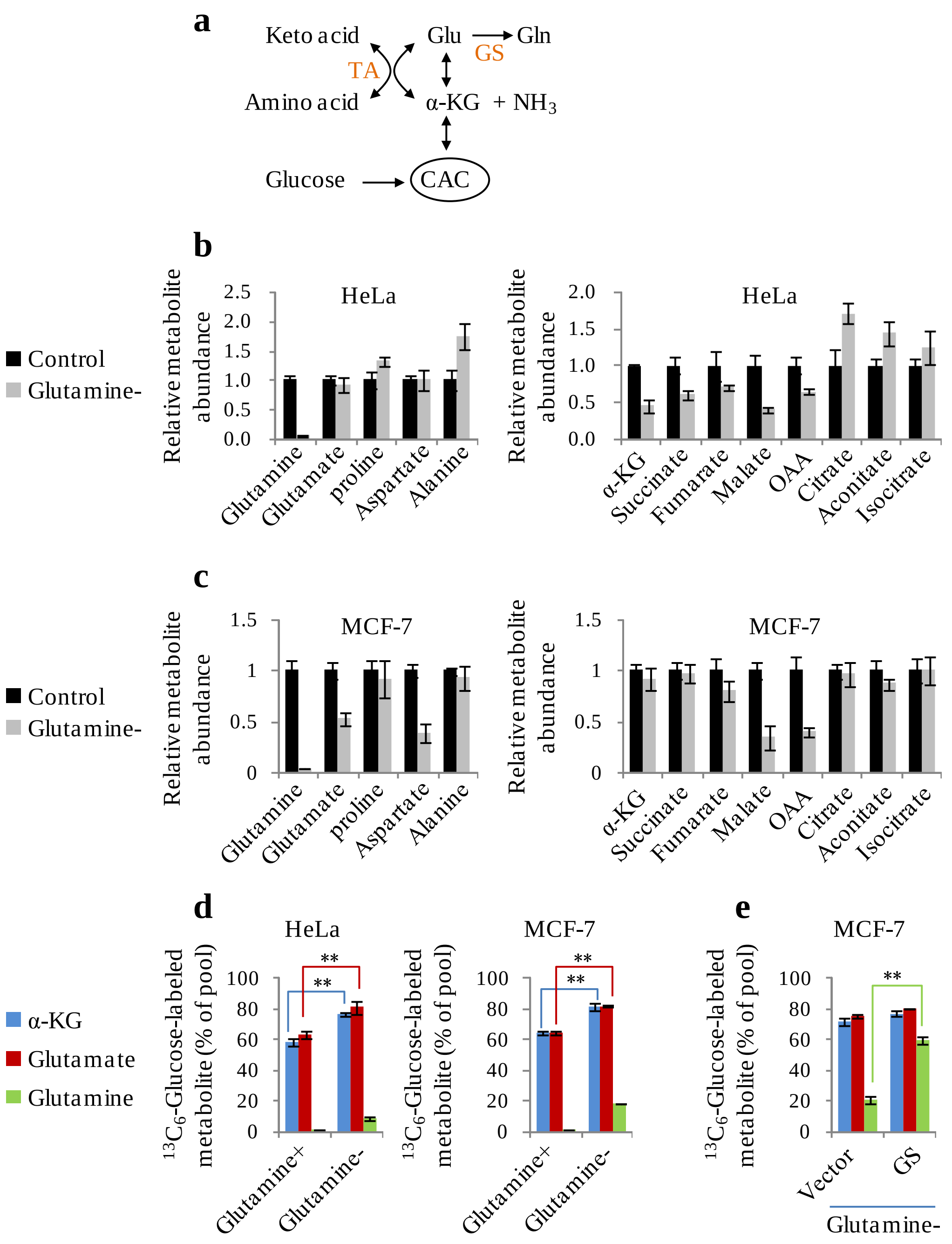

Supplementary Figure 2. Glutamine biosynthesis depends on glutamine synthetase. (a) A schematic to show the metabolic connection between glucose and glutamine. GS, glutamine synthetase; TA, transaminases. (b,c) Relative metabolite abundance in HeLa and MCF-7 cells cultured in the absence or presence of glutamine (2 mM) for 8h. (d) The labeled fraction of α-ketoglutarate, glutamate and glutamine in HeLa and MCF-7 cells cultured with the glutamine-contained or -free medium containing 10 mM of $^{13}C_6$-glucose for 8h. (e) The labeled fraction of α-ketoglutarate, glutamate and glutamine in MCF-7 cells expressing GS or empty vector cultured with the glutamine-free medium containing 10 mM of $^{13}C_6$-glucose for 8h. All cultures were supplied with 10% dialyzed serum. Values are the means ± SEM of three independent experiments. *p < 0.05, **p < 0.01 (Student's t-test).

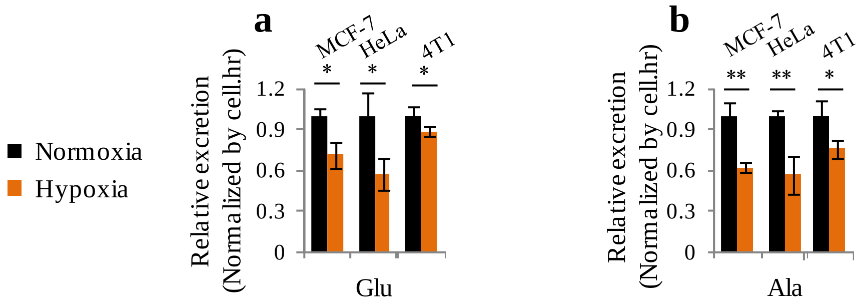

Supplementary Figure 3. Decreased excretion of glutamate and alanine under hypoxia. (a,b) Relative glutamate and alanine excretion from MCF-7, HeLa and 4T1 cells cultured under hypoxia and normoxia for 8h. All cultures were supplied with 10% dialyzed serum. Values are the means ± SEM of three independent experiments. *$p < 0.05$, **$p < 0.01$ (Student's *t*-test).

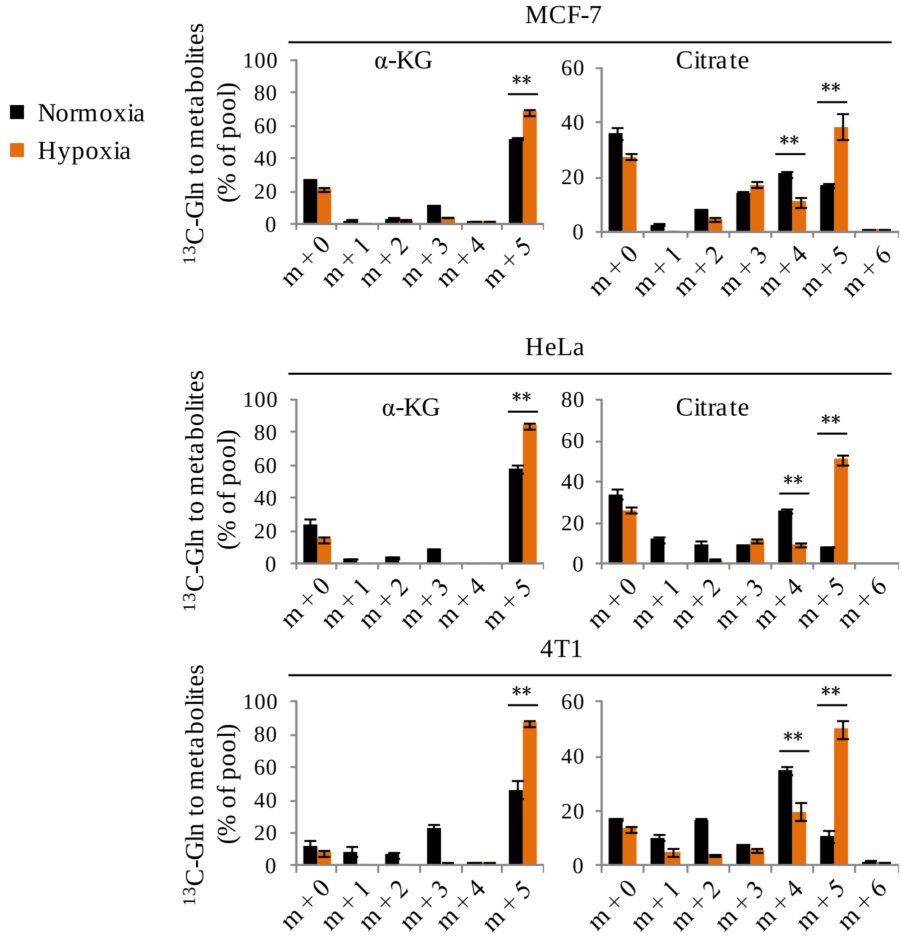

Supplementary Figure 4. Metabolic flux of glutamine-carbon to α-ketoglutarate and citrate under normoxia and hypoxia. Mass isotopomer analysis of cellular α-ketoglutarate and citrate in MCF-7, HeLa and 4T1 cells cultured with the medium containing 1 mM of $^{13}C_5$-glutamine for 8h under hypoxia or normoxia. Values are the means ± SEM of three independent experiments. *$p < 0.05$, **$p < 0.01$ (Student's *t*-test).

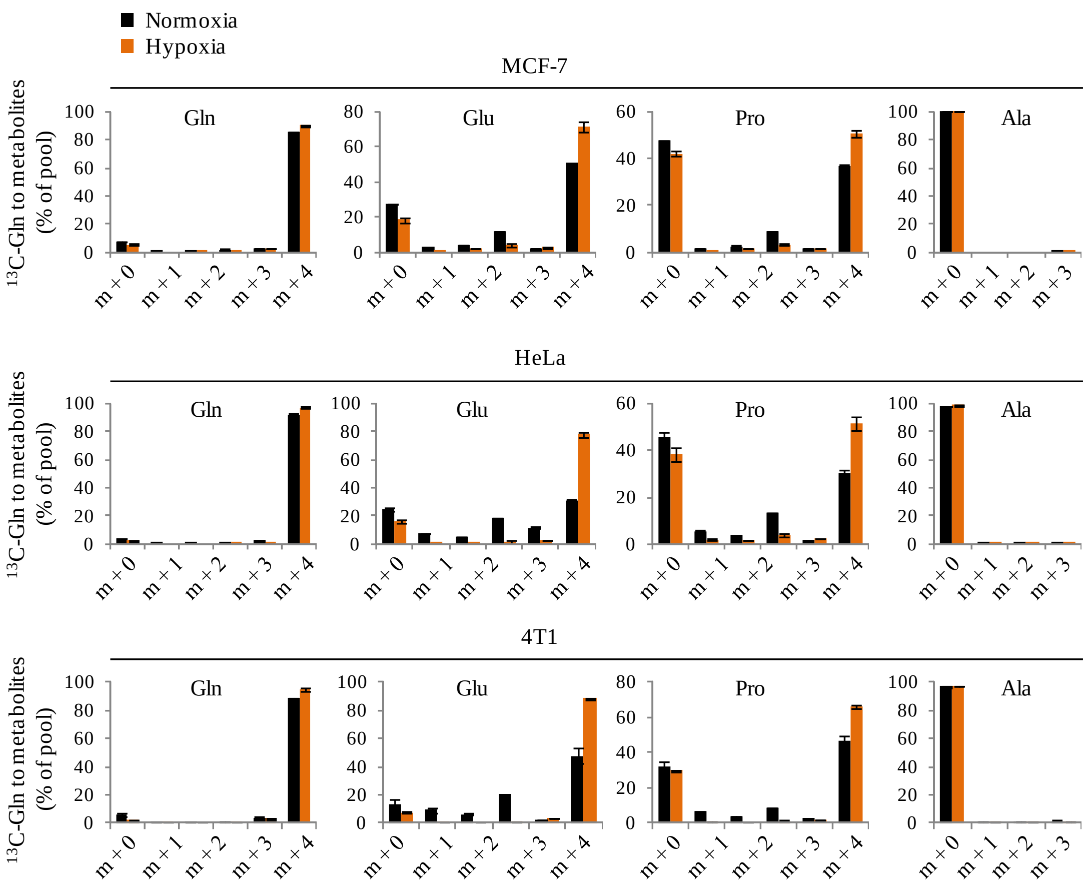

Supplementary Figure 5. Metabolic flux of glutamine-carbon to amino acids under normoxia and hypoxia. Mass isotopomer analysis of cellular amino acids in MCF-7, HeLa and 4T1 cells cultured with the medium containing 1 mM of $^{13}C_5$-glutamine for 8h under hypoxia or normoxia. Values are the means ± SEM of three independent experiments. *$p < 0.05$, **$p < 0.01$ (Student's *t*-test).

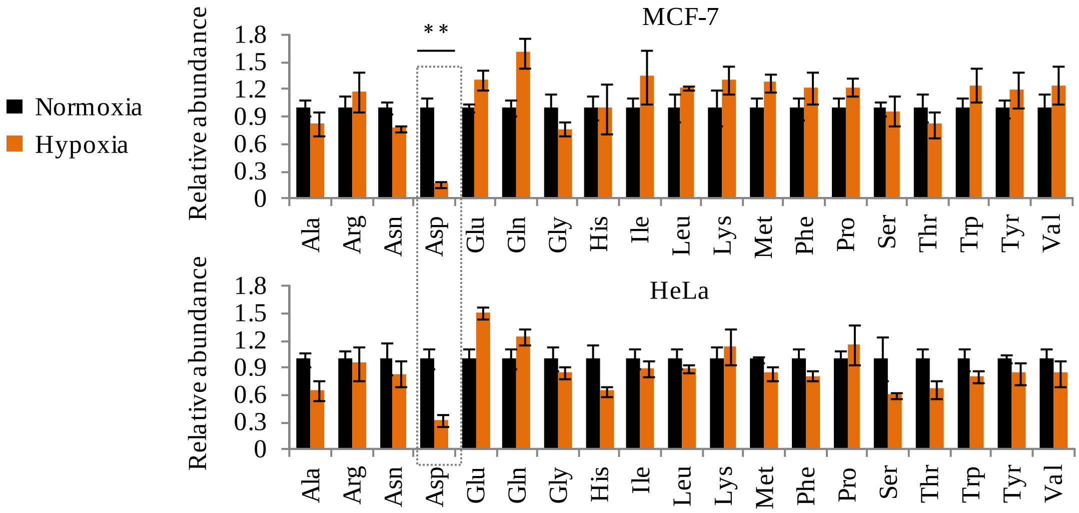

Supplementary Figure 6. The level of cellular amino acids under normoxia and hypoxia. Relative cellular amino acids in MCF-7 and HeLa cells cultured under hypoxia and normoxia for 8h. All cultures were supplied with 10% dialyzed serum. Values are the means ± SEM of three independent experiments. *p < 0.05, **p < 0.01 (Student's t-test).

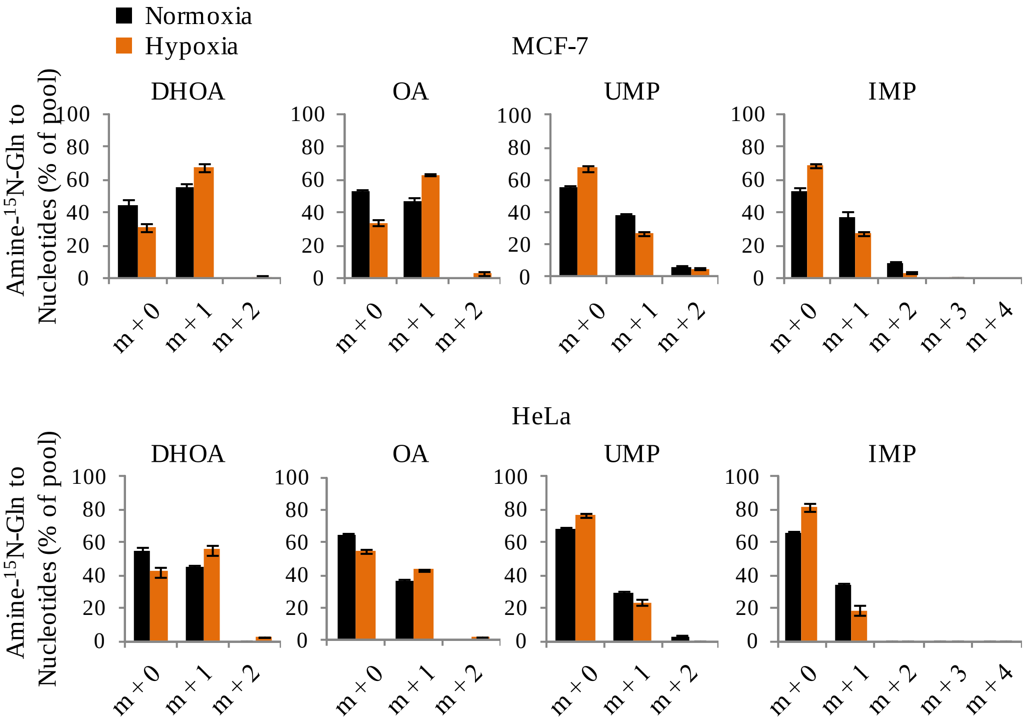

Supplementary Figure 7. Metabolic flux of glutamine-amine nitrogen in the nucleoside biosynthesis under hypoxia and normoxia. Mass isotopomer analysis of cellular dihydroorotate, orotate, UMP and IMP in MCF-7 and HeLa cells cultured with the medium containing 1 mM of amine-^{15}N-glutamine for 8h under hypoxia or normoxia. All cultures were supplied with 10% dialyzed serum. Values are the means ± SEM of three independent experiments. *$p < 0.05$, **$p < 0.01$ (Student's *t*-test).

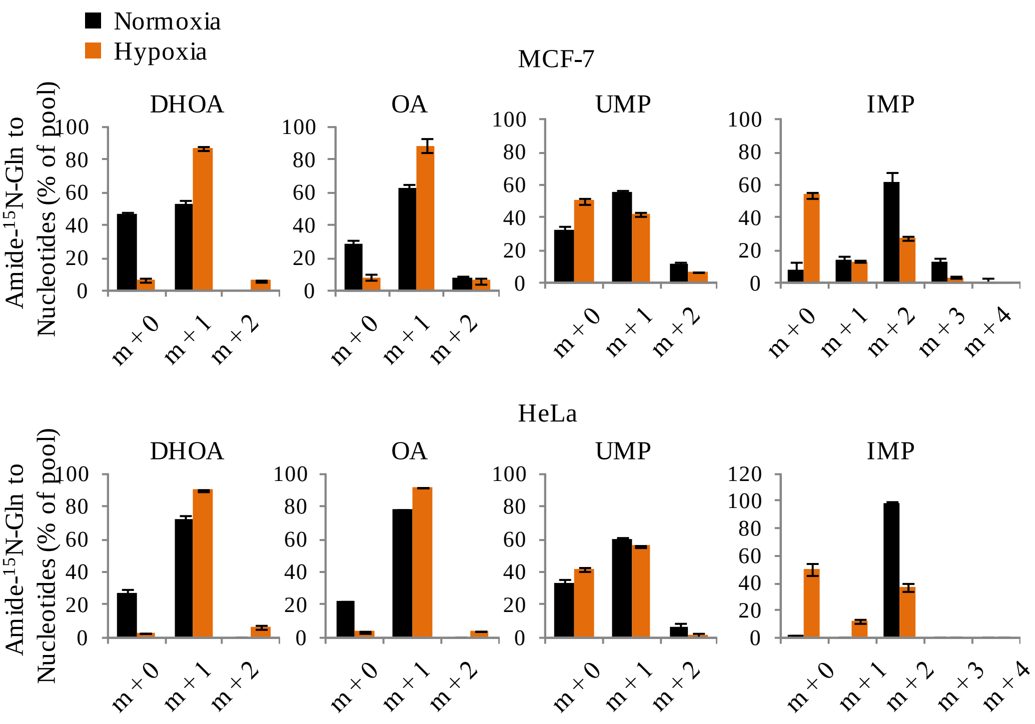

Supplementary Figure 8. Metabolic flux of glutamine-amide nitrogen in the nucleoside biosynthesis under hypoxia and normoxia. Mass isotopomer analysis of cellular dihydroorotate, orotate, UMP and IMP in MCF-7 and HeLa cells cultured with the medium containing 1 mM of amide-^{15}N-glutamine for 8h under hypoxia or normoxia. All cultures were supplied with 10% dialyzed serum. Values are the means ± SEM of three independent experiments. *$p < 0.05$, **$p < 0.01$ (Student's *t*-test).

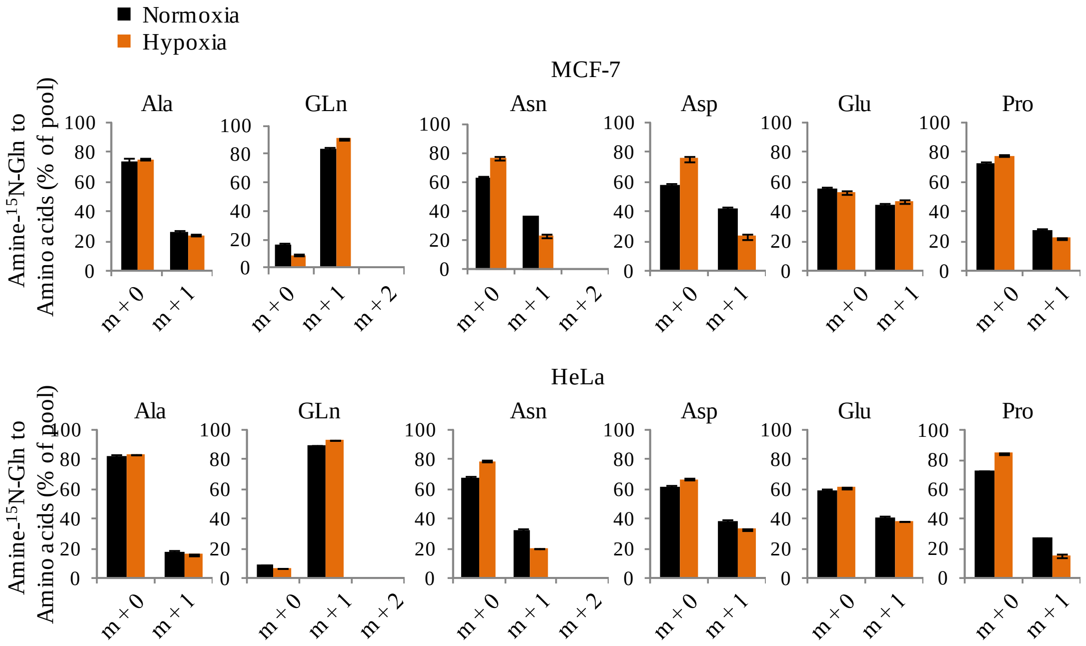

Supplementary Figure 9. Metabolic flux of glutamine-amine nitrogen to amino acids under hypoxia and normoxia. Mass isotopomer analysis of cellular amino acids in MCF-7 and HeLa cells cultured with the medium containing 1 mM of amine-^{15}N-glutamine for 8h under hypoxia or normoxia. All cultures were supplied with 10% dialyzed serum. Values are the means ± SEM of three independent experiments. *p < 0.05, **p < 0.01 (Student's t-test).

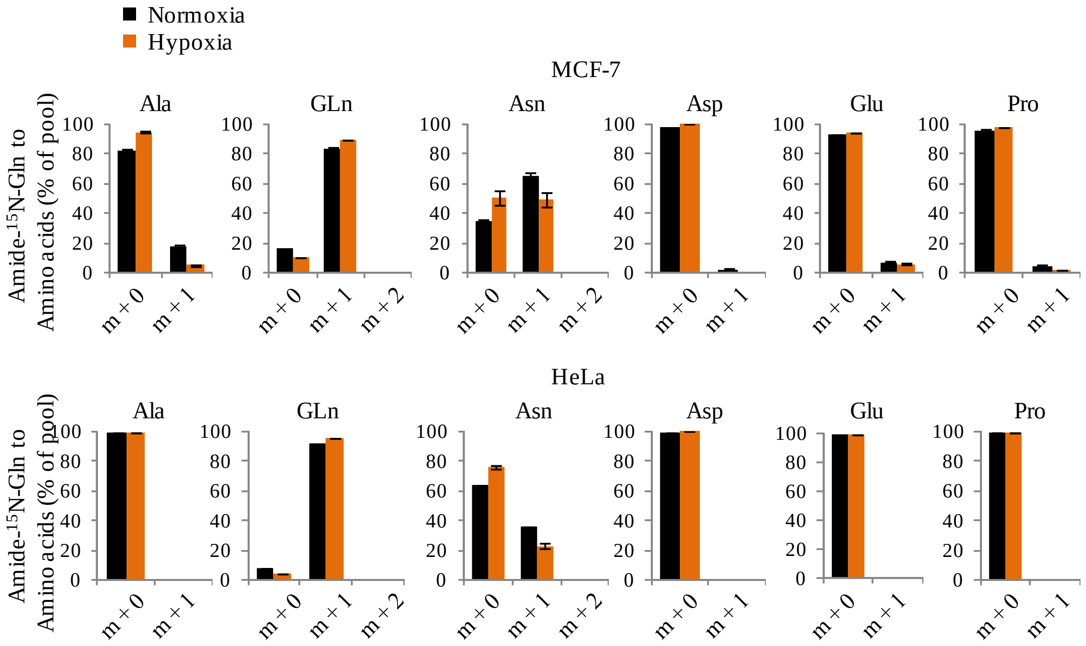

Supplementary Figure 10. Metabolic flux of glutamine-amide nitrogen to amino acids under hypoxia and normoxia. Mass isotopomer analysis of cellular amino acids in MCF-7 and HeLa cells cultured with the medium containing 1 mM of amide-^{15}N-glutamine for 8h under hypoxia or normoxia. All cultures were supplied with 10% dialyzed serum. Values are the means ± SEM of three independent experiments. *p < 0.05, **p < 0.01 (Student's t-test).

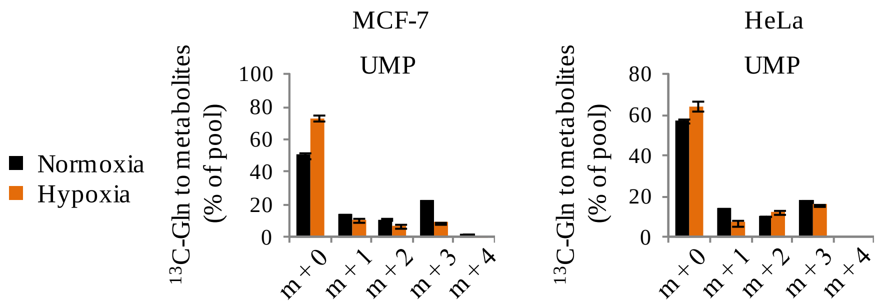

Supplementary Figure 11. Metabolic flux of glutamine-carbon to UMP under normoxia and hypoxia. Mass isotopomer analysis of cellular UMP in MCF-7 and HeLa cells cultured with the medium containing 1 mM of $^{13}C_5$-glutamine for 8h under hypoxia or normoxia. All cultures were supplied with 10% dialyzed serum. Values are the means ± SEM of three independent experiments. **p* < 0.05, ***p* < 0.01 (Student's *t*-test).

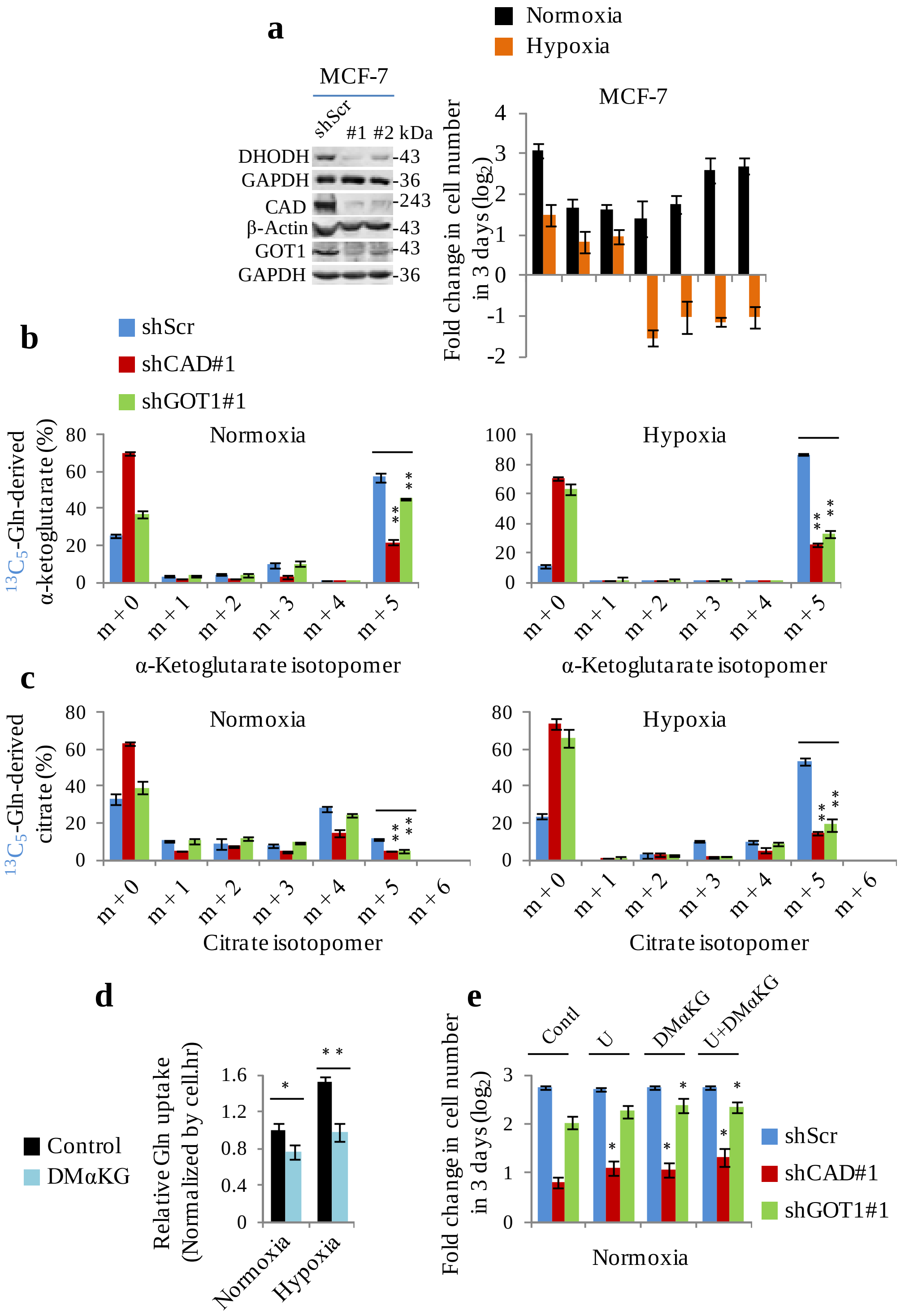

Supplementary Figure 12. Effects of knockdown of CAD and GOT1 on glutamine metabolism. (a) Proliferation of MCF-7 cells with or without knockdown of DHODH, CAD and GOT1 cultured under hypoxia and normoxia for 3 days. Values are the means ± SEM of triplicate experiments. Western blot to validate the knockdown of DHODH, CAD and GOT1. **(b,c)** Mass isotopomer analysis of α-ketoglutarate and citrate in HeLa/shScramble, HeLa/shCAD and HeLa/shGOT1 cells cultured with the medium containing 1 mM of $^{13}C_5$-glutamine under hypoxia or normoxia for 8h. Values are the means ± SEM of triplicate experiments. **(d)** Relative glutamine uptake in MCF-7, HeLa and 4T1 cells cultured under

hypoxia and normoxia for 8h in the presence or absence of 2 mM dimethyl α-ketoglutarate (DMαKG). **(e)** Proliferation of HeLa/shScramble, HeLa/shCAD and HeLa/shGOT1 cells cultured in the normal condition for 3 days in the presence or absence of 100 μM uridine and/or 2 mM DMαKG. Values are the means ± SEM of triplicate experiments. All cultures were supplied with 10% dialyzed serum. *$p < 0.05$, **$p < 0.01$ (Student's *t*-test).

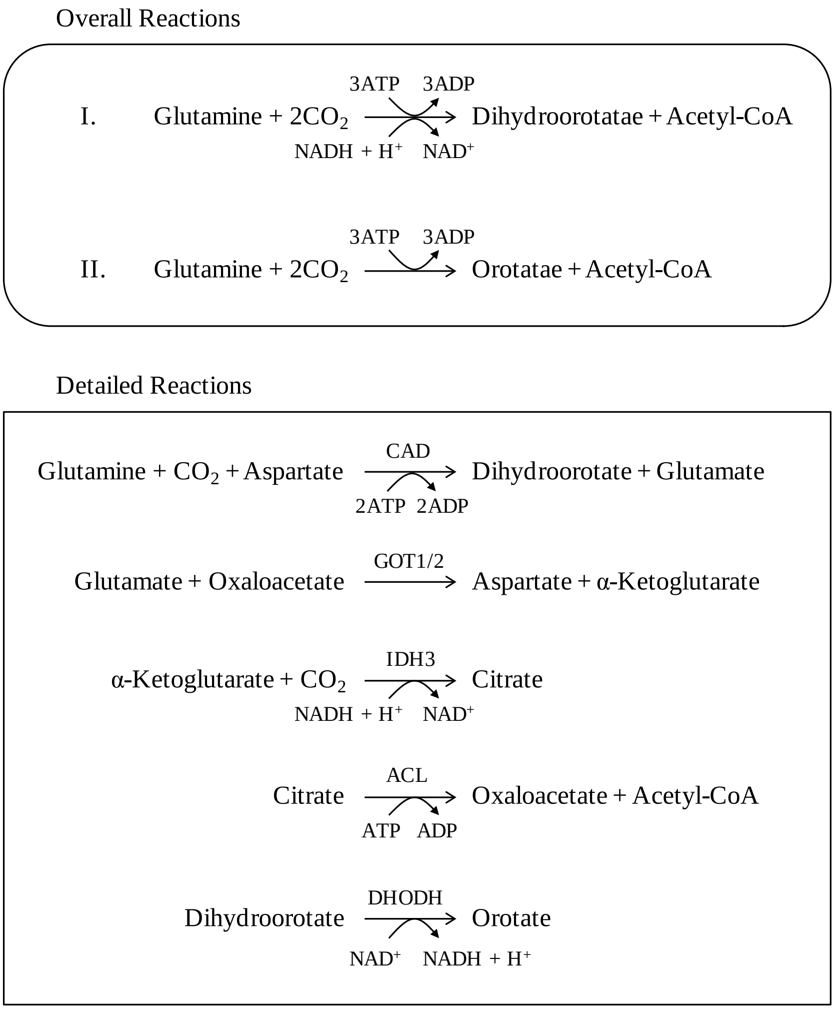

Supplementary Figure 13. Metabolic conversions of glutamine to dihydroorotate or orotate. The overall reactions (the upper panel) were summarized based on the detailed reactions (the bottom panel).

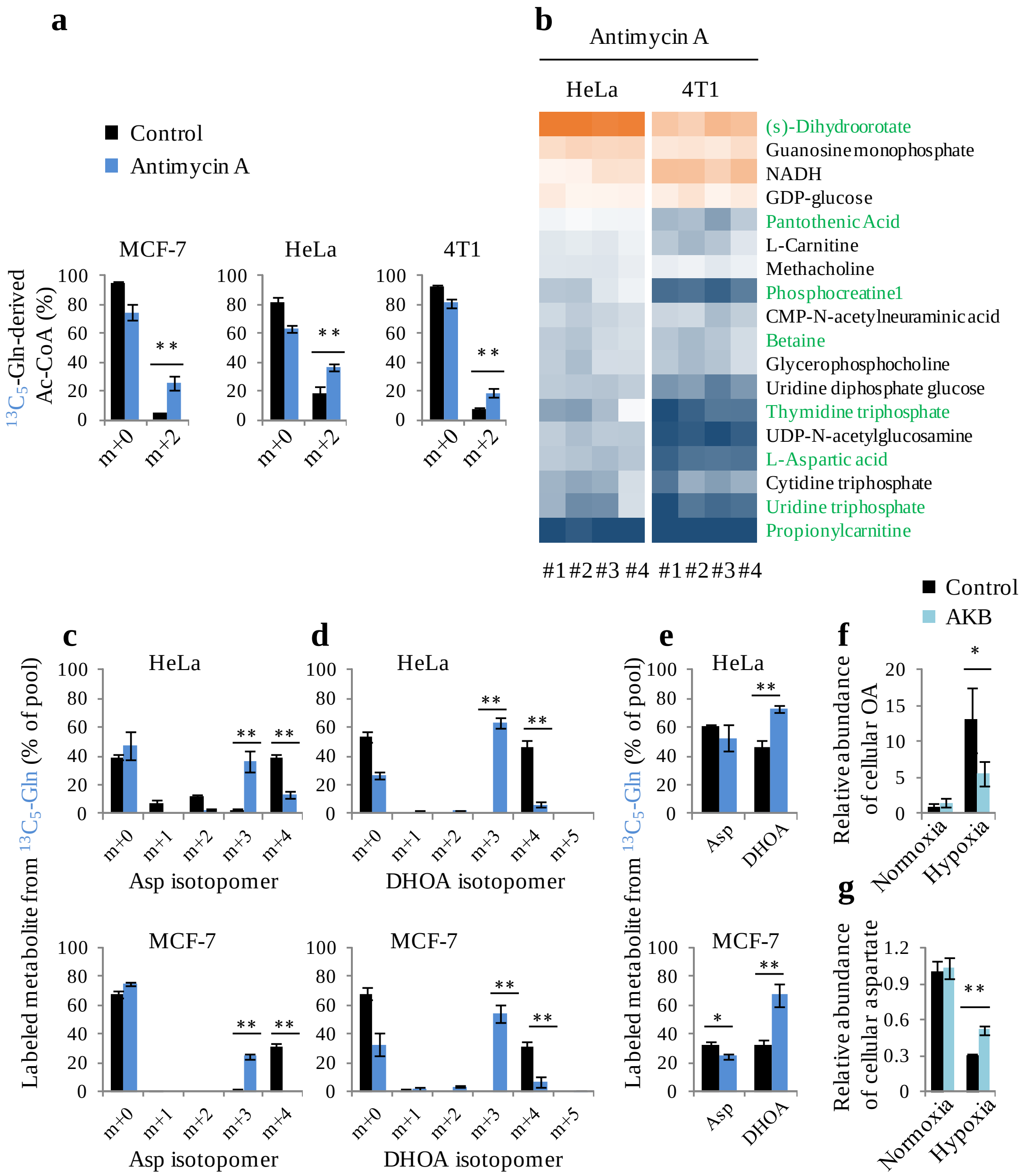

Supplementary Figure 14. Effects of ETC inhibition by antimycin A on cellular metabolism. (a) Mass isotopomer analysis of acetyl-CoA in MCF-7, HeLa and 4T1 cells cultured with the medium containing 1 mM of $^{13}C_5$-glutamine in the absence or presence of 1 μM of antimycin A. **(b)** Heatmap of N-contained metabolites in HeLa and 4T1 cells significantly (n=4, p < 0.05) affected by antimycin A treatment for 8h. Cellular metabolites were measured by LC-MS-based metabolomics. Metabolites with green color were also covered by hypoxia in Figure 3A. **(c,d)** Mass isotopomer analysis of aspartate and dihydroorotate in MCF-7 and HeLa cells cultured with the medium containing 1 mM of $^{13}C_5$-glutamine in the absence or presence of 1 μM of antimycin A. **(e)** The $^{13}C_5$-labeled fraction of metabolites in MCF-7 and HeLa cells cultured with the medium containing 1 mM of $^{13}C_5$-glutamine in the absence or presence of 1 μM of antimycin A. **(f,g)** The relative abundance of cellular orotate and asparatate in HeLa cells cultured under hypoxia and normoxia for 8h in the presence or absence of 1 mM α-ketobutyrate. All cultures were

supplied with 10% dialyzed serum. Values are the means ± SEM of three independent experiments. $*p < 0.05$, $**p < 0.01$ (Student's *t*-test).

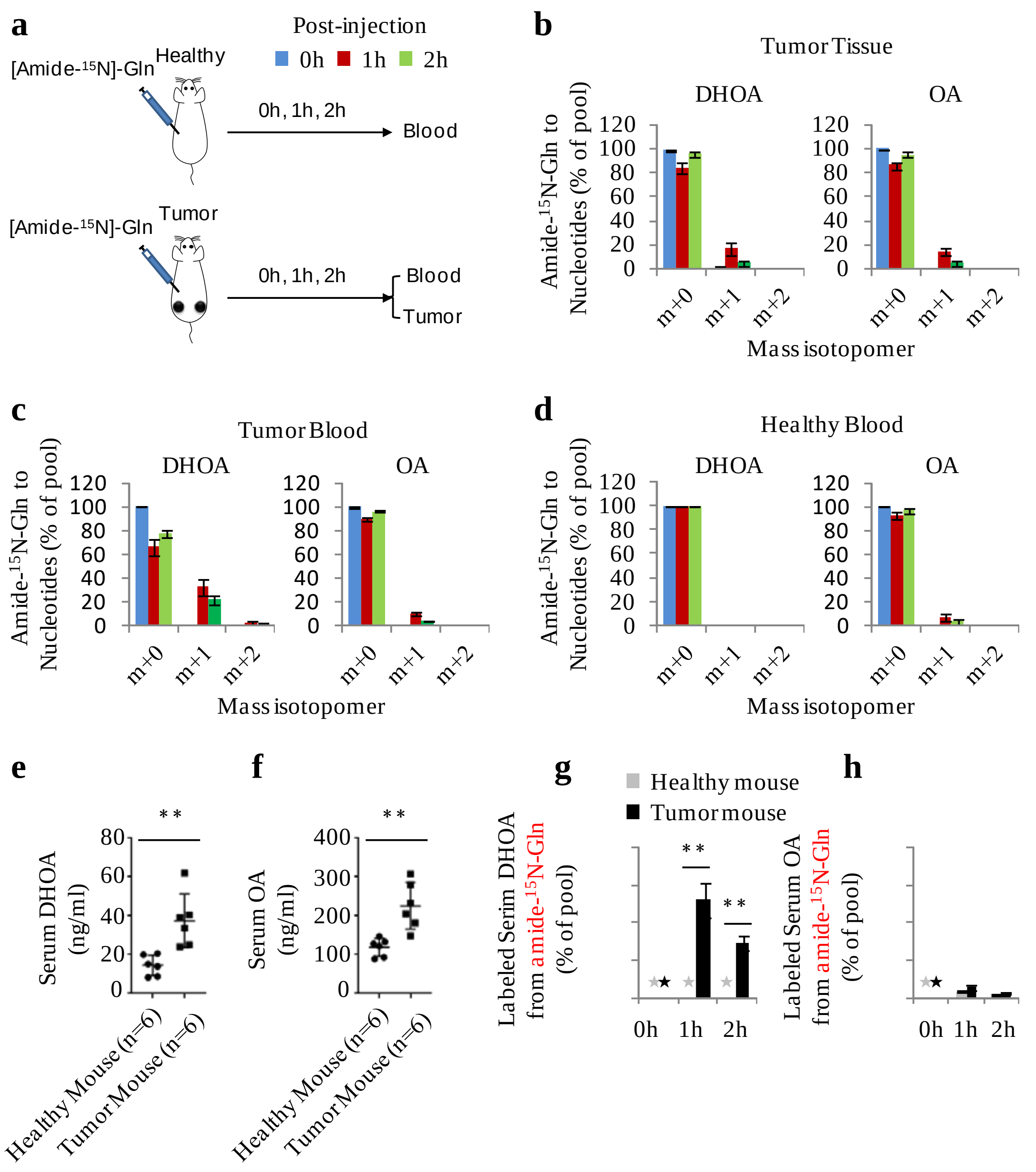

Supplementary Figure 15. Glutamine-derived dihydroorotate and orotate in tumor-bearing Mice. (a) A schematic to show how to do the animal experiments. Healthy and HeLa-derived tumor-bearing nude mice were intraperitoneally injected with 5 mmol kg^{-1} of amide-^{15}N-glutamine for 0h, 1h or 2h, and the blood and tumor tissue samples were prepared as described in the Method section. **(b)** Mass isotopomer analysis of dihydroorotate and orotate of tumor tissues from HeLa-derived tumor-bearing mice. Values are the means ± SEM of data from three mice. **(c,d)** Mass isotopomer analysis of blood dihydroorotate and orotate in healthy and HeLa-derived tumor-bearing nude mice. Values are the means ± SEM of data from three mice. **(e,f)** Serum dihydroorotate and orotate in healthy and 4T1-derived tumor-bearing nude mice. **(g,h)** The ^{15}N-labeled fraction of blood dihydroorotate and orotate in healthy and 4T1-derived tumor-bearing nude mice intraperitoneally injected with 5 mmol kg^{-1} of amide-^{15}N-glutamine for 1h or 2h. Values are the means ± SEM of data from three mice. *$p < 0.05$, **$p < 0.01$ (Student's *t*-test).

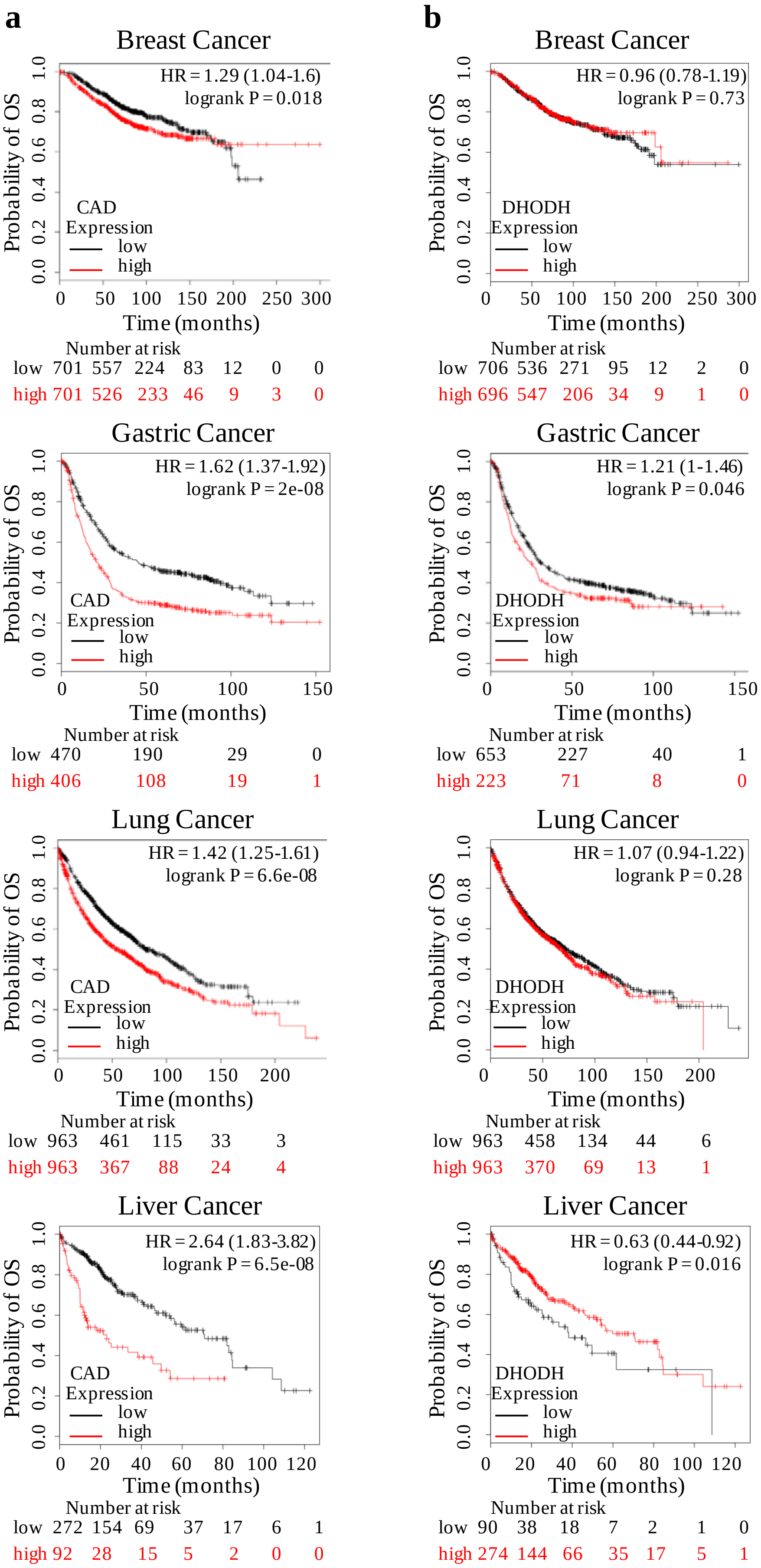
a
b
Breast Cancer
HR = 1.29 (1.04-1.6)
logrank P = 0.018
CAD
Expression
low
high
Probability of OS
Time (months)
Number at risk
low 701 557 224 83 12 0 0
high 701 526 233 46 9 3 0
Breast Cancer
HR = 0.96 (0.78-1.19)
logrank P = 0.73
DHODH
Expression
low
high
Number at risk
low 706 536 271 95 12 2 0
high 696 547 206 34 9 1 0
Gastric Cancer
HR = 1.62 (1.37-1.92)
logrank P = 2e-08
CAD
Expression
Number at risk
low 470 190 29 0
high 406 108 19 1
Gastric Cancer
HR = 1.21 (1-1.46)
logrank P = 0.046
DHODH
Expression
Number at risk
low 653 227 40 1
high 223 71 8 0
Lung Cancer
HR = 1.42 (1.25-1.61)
logrank P = 6.6e-08
CAD
Expression
Number at risk
low 963 461 115 33 3
high 963 367 88 24 4
Lung Cancer
HR = 1.07 (0.94-1.22)
logrank P = 0.28
DHODH
Expression
Number at risk
low 963 458 134 44 6
high 963 370 69 13 1
Liver Cancer
HR = 2.64 (1.83-3.82)
logrank P = 6.5e-08
CAD
Expression
Number at risk
low 272 154 69 37 17 6 1
high 92 28 15 5 2 0 0
Liver Cancer
HR = 0.63 (0.44-0.92)
logrank P = 0.016
DHODH
Expression
Number at risk
low 90 38 18 7 2 1 0
high 274 144 66 35 17 5 1

Supplementary Figure 16. The relationship between CAD and DHODH and the overall survival time in cancer patients. (a,b) Correlation of CAD (**a**) or DHODH (**b**) with the overall survival in patients of breast cancers, lung cancers, gastric cancer and liver cancers were organized based on an online source: http://kmplot.com/analysis/.

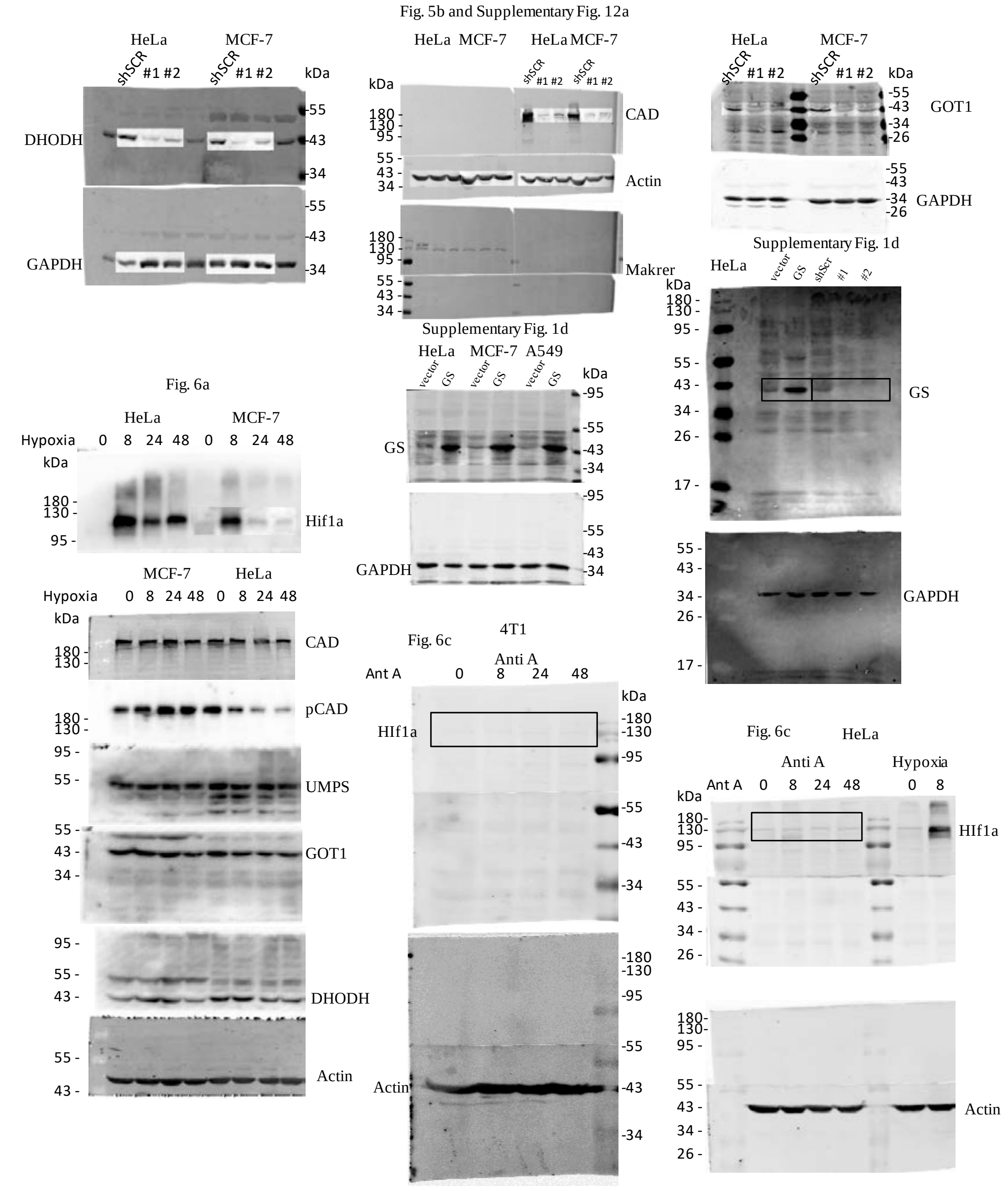

Supplementary Figure 17. Uncropped Western blot gels.
